# The role of total fats, saturated/unsaturated fatty acids and cholesterol content in chicken meat as cardiovascular risk factors

**DOI:** 10.1186/1476-511X-13-42

**Published:** 2014-03-03

**Authors:** Dragan Milićević, Danijela Vranić, Zoran Mašić, Nenad Parunović, Dejana Trbović, Jelena Nedeljković-Trailović, Zoran Petrović

**Affiliations:** 1 Institute of Meat Hygiene and Technology, Kaćanskog 13, 11040 Belgrade, Serbia; 2 Scientific Veterinary Institute “Novi Sad” Rumenički put 20, 21000 Novi Sad, Serbia; 3 Department of Animal nutrition and botany, Faculty of Veterinary Medicine - University of Belgrade, 18 Bulevar oslobođenja, 11000 Belgrade, Serbia

**Keywords:** Chicken meat, Cholesterol, Fatty acids, Cardiovascular risk

## Abstract

**Background:**

The objective of the study was to present information about the chemical composition, the fatty acids profile, and cholesterol content of chicken meat in order to investigate the impact of chicken meat consumption on cardiovascular risk in the general population.

**Methods:**

A total of 48 6-wk-old broiler chickens broilers from two farms in June to November of 2012, and February of 2013, were used in this trial. Total lipid content was determined by extraction of fat by petrol ether (Soxhlet) after acid hydrolysis of samples. Fatty acids were determined by capillary gas chromatography. Cholesterol determination was performed by using HPLC/PDA system.

**Results:**

The results indicate that the total free cholesterol content in raw breast and drumstick of chickens was in the range of 37,41–79,9 mg/100 g and 48,35-99,5 mg/100 g, respectively. The main fatty acids identified in all cuts were C18:1c9, C18:2n6, C16:0, C18:0, and C16:1. Decreasing the dietary n-6/n-3 clearly decreased the content in breast and drumstick muscle of C18:2n6, C18:3n3, and C20: 3n6, but increased that of C16:0, C18:0, and C20:2. Also, the major saturated fatty acid (SFA) (C16:0 and C18:0) was significantly differ among the four treatments.

**Conclusion:**

Our study shows that dietary fat and fatty acid composition influence the concentrations of total cholesterol content, total fat content, and fatty acid composition in broiler muscle. This information will aid in determining the burden of chicken meat as a cardiovascular risk factors disease and act as a planning tool for public-health Programmes.

## Background

In the human diet, meat is seen as a major source of fat, and especially of saturated fatty acids (SFAs), which have been implicated in diseases associated with modern life, mostly in developed countries. Coronary heart disease and arteriosclerosis are among the most important causes of human mortality, and are strongly associated with dietary intake of cholesterol and saturated fatty acids [1,2]. In addition, a strong relationship has been demonstrated between cellular cholesterol concentration and Alzheimer’s disease [3]. The World Health Organization [4] recommends that the daily fat intake be reduced to 30% of the total energy intake, and that saturated fats should be limited to 10% of this caloric intake. It is also advised that cholesterol intake should not exceed 300 mg per day.

Dietary intake of unsaturated fatty acids (UFA) has been shown to reduce the risk of cardiovascular disease (CVD) and possibly the incidence of some cancers, asthma and diabetes among other conditions. At the same time, the recommended ratio of polyunsaturated fatty acids (PUFAs) to SFAs (P/S) should be above 0.4, with the normal P/S ratio of meat at around 0.1 [5]. The ratio of n-6/n-3 PUFAs is considered to be a risk factor in cancers and coronary heart disease, and it is recommended that this ratio be less than 4.0 [6]. Achieving a better balance of fatty acids in the diet, by decreasing intakes of cholesterol and saturated fats, is therefore seen as an important and effective strategy by which to reduce the incidence of these diseases. Therefore, the knowledge on the cholesterol content in food is important, especially in poultry and fish meat, because the consumption of these foods is currently increasing based on the recommendations of healthy nutrition.

The objective of the present study was to present information about the cholesterol content fat content and n - 6/n - 3 ratio of chicken meats in order to estimate whether the expected health effects of the former are potentially the same as those of the latter, especially in relation to cardiovascular diseases. In an array of data regarding cholesterol content and fatty acid composition of meat, the new contribution of the study can be seen in the following aspects: cholesterol content and fatty acid composition of the animal tissues can be influenced by the composition of the feed mixtures, especially by the ratio of polyunsaturated fatty acids [7].

## Results

### Carcass quality

The physicochemical properties and quality characteristics of chicken meat, reared under standard condition are presented in Table 1.

**Table 1:** **Physicochemical properties and quality characteristics of chicken meat between farms during two period of investigation**$\left(\overset{¯}{X}\pm \;\mathrm{Sd}\right)$

**Parameter**		**Tretman**			
		**I**^**a**^	**II**^**a**^	**I**^**b**^	**II**^**b**^
Average live weight (g)		2190^a^	1470^b, a^	2240^b, e^	2220^b, f^
Age at slaughter (d)		38	38	39	38
Carcass weight (g)		1279,45 ±174,02	1223,3±116,38	1764,87±227,83	1400,70±130,01
Weight (g)	Breast	452,5±80,17	427,70±53,44	647,95±78,70	470,62±61,15
	Drumstick	389,89±51,19	392,9±39,20	536,40±88,41	476,78±48,20
pH, Ultimate	Breast	5,4±0,20^a^	5,90±0,11^b^	5,88±0,18^b^	5,84±0,14^b^
	Drumstick	5,71±0,30^a^	6,44±0,17^b^	6,45±0,26^b^	6,51±0,09^b^
Water-holding capacity (%)	Breast	30,53±7,27^e^	29,44±10,95^c^	36,58±7,70^d^	42,78±10,93^f, d^
	Drumstick	17,04±5,60^a^	26,50±9,81^b, c^	31,60±7,82^b^	37,94±9,83^b, d^
Moisture (%)	Breast	71,25 ± 2,75	70,74±3,79^a^	71,48±2,58	74,29±1,32^b^
	Drumstick	69,50±1,40^a^	71,765±1,78	71,13±0,63^b^	72,29±2,81
Protein (%)	Breast	21,18±0,96^e^	21,65±0,75	22,18±0,25^f^	22,29±0,71
	Drumstick	17,95±0,956	18,68±0,96	18,41±0,95	17,92±0,61
Lipids (%)	Breast	5,53±0,61^a^	3,05±1,11^b, c^	4,19±1,15^b, d, e^	2,61±0,77^b, f^
	Drumstick	9,63±0,63^a^	5,19±0,45^b, a^	9,85±1,98^b^	8,16±2,25^b^
Ash (%)	Breast	0,99±0,13^a^	1,16±0,09^b, e^	1,04±0,09^f, a^	1,29±0,11^b, f^
	Drumstick	0,92±0,05^f^	1,026±0,05^f, a^	0,91±0,06^b, a^	1,01±0,07^f, b^
Fe total (mg/kg)	Breast	4,06±1,36	4,34±0,55^d^	3,94±0,42^c^	4,29±0,31^d^
	Drumstick	7,88±1,41	7,45±0,98	8,42±1,46	8,12±0,68

n = 12 per Tretman, ^a:b^p<0,001, ^c:d^p<0,05, ^e:f^p<0,01, (Tretman-Farm I and II), a-summer, b- winter.

The results from physicochemical properties and quality characteristics in the current study are consistent with previous results from the same laboratory. Diets had no effect on meat sensory attributes (data not presented). Comparing the meat quality characteristics among treatments (farms), significant differences were found for all parameters except for Fe content in drumstick. Dark meat was found to contain slightly more than twice as much Iron as white meat (8.42 ± 1.46 vs 3.94 ± 0.42 mg/kg), respectively. The means for total iron reported in this study for both white (breasts) and dark (drumsticks) chicken meat, are similar to the 5 and 10 mg/kg reported by McCance et al. [8]. The pH at 24 hours post-mortem on the treatment I in summer period had a significantly lower (P<0,001) pH than birds from other farms. In Breast pH was 5.40 ± 0.20 versus 5.90 ± 0.11, 5.88 ± 0.18 versus 5.84 ± 0.14 while in Drumstick was 5.71 ± 0.30 versus 6.44 ± 0.17, 6.45 ± 0.26 versus 6.51 ± 0.09. These results show that the pH values of pale samples were 5.40 ± 0.20 and 5.71 ± 0.30, respectively. Meat products with low pH values are associated with poor water-holding capacity and meat color [9,10]. These results are in agreement with those reported by Vimini, (1996) [11] and Barbut (1998) [12] in which the incidence of PSE-quality broiler breast meat varies from 2 to 20%, depending on the environmental conditions. In the current study, a higher ultimate pH value was found in drumstick in the three farms and were 6.44 ± 0.17, 6.45 ± 0.26 and 6.51 ± 0.09, respectively. Water-holding capacity, reported as percentage expressible juice, was 30.53 ± 7.27% and 17.04 ± 5.60% of the PSE samples, 29.44 ± 10.95-42.78 ± 10.93% of the normal samples, and 26.50 ± 9.81–37.94 ± 9.83% of the dark samples. There were very low level of negative or positive correlations between pH and WHC (-0.280 to 0,245), except in one case where moderate positive correlation between pH and WHC (0.401) was recorded.

### Cholesterol concentration

**Figure 1:**
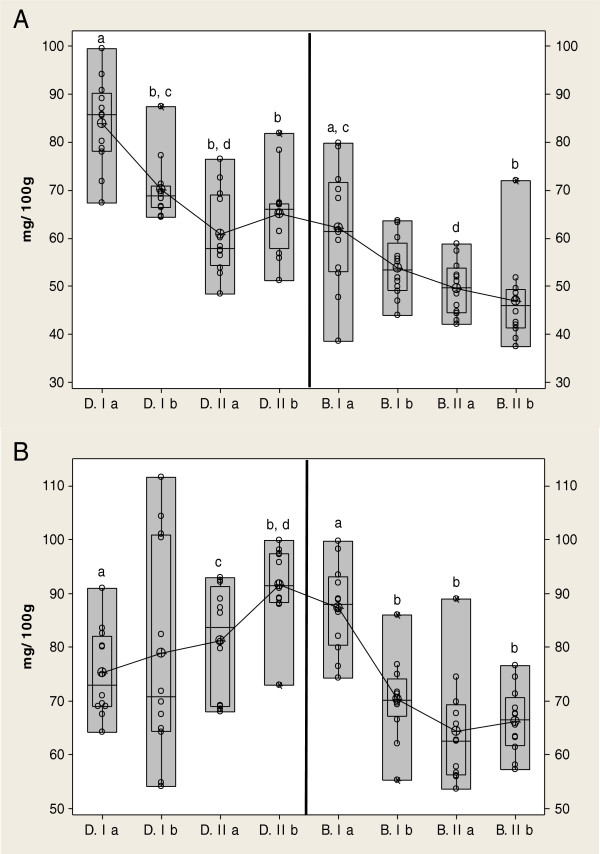
**Content of cholesterol in Raw (A) and Heat (B) processed Breast and Drumstick muscle between farms during two period of investigation (D.-Drumstick, B.- Breast muscle, I, II-farm, a Summer, b Autumn/winter period) **^**a:b**^**p<0,001 **^**b:c**^**p<0,01.**

The cholesterol concentrations of raw drumstick and breast muscles are presented in Figure 1A.

Breast muscle showed lower values, possibly because of its lower fat content. The cholesterol content in Breast meat in the present study varied from 37.41 to 79.9 mg/100 g, while in Drumstick varied from 48.35 to 99.5 mg/100 g in depend on treatments. As shown in this study, the magnitude of variation of the cholesterol contents in muscles appears to be high (Figure 1A). Thus, significant differences (P<0.001) in content of cholesterol were observed among farms. The cholesterol levels in chicken meat values presented in this paper are generally within the ranges of the published data [13-17]. In general, raw poultry meat has approximately 27 to 90 mg cholesterol/100 g and cooked poultry meat contains around 59 to 154 mg/100 g [14,16]. In addition, the difference between chicken breast fillet and leg and thigh is of the same magnitude seen in other studies.

### Fatty acid composition of breast and drumsticks muscle

The fatty acid profile of breast and drumsticks muscle are presented in Tables 2 and 3.

**Table 2:** **Fatty Acids Composition (mg/100 g) of Breast muscle**$\left(\bar{X}\pm \;\mathrm{Sd}\right)$

**Fatty acids (range)**	**Tretman**			
	**I**^**a**^	**II**^**a**^	**I**^**b**^	**II**^**b**^
C14:0	0, 32±0,014^**e**, **a**^	0,35±0,02^**f**^	0,34±0,03	0,43±0,02^**b**^
C15:0	0,05±0,004^**a**^	0,06±0,01^**b, e, a**^	0,05±0,01^**f, a**^	0,13±0,03^**b**^
C16:0	23,16±0,48^**a, e**^	24,36±0,51^**b, a**^	22,26±0,61^**f, b, a**^	26,81±0,79^**b**^
C16:1	4,34±0,35^**e, a**^	3,81±0,49^**f**^	3,54±0,14^**b**^	3,85±0,72
C17:0	0,09±0,01^**c, e, a**^	0,10±0,01^**d, a**^	0,10±0,01^**f, a**^	0,15±0,02^**b**^
C18:0	6,04±0,37^**a**^	7,72±0,58^**b, a, c**^	6,68±0,22^**b, a**^	8,76±0,78^**b, f**^
C18:1c-9	39,56±1,21^**c, a**^	38,06±1,40^**d, c**^	37,12±1,26^**b, d, c**^	38,38±1,39^**d**^
C18:2n6	23,22±1,14^**e, a**^	21,87±1,21^**f, a**^	25,83±0,87^**b, a**^	14,89±1,93^**b**^
C18:3n6	0,19±0,05^**a**^	0,22±0,04^**e, a**^	0,16±0,03^**f, a**^	0,01±0,03^**b**^
C18:3n3	1,31±0,06^**a, e**^	1,09±0,08^**b, a**^	1,48±0,16^**f, b, a**^	0,51±0,09^**b**^
C20:0	0,07±0,03^**c**^	0,09±0,02^d**, e**^	0,09±0,02^**c**^	0,06±0,02^**f, d**^
C20:1	0,49±0,03^**e, a**^	0,46±0,01^**f, c, a**^	0,38±0,09^**b, d**^	0,39±0,03^**b**^
C20:2	0,23±0,04^**a**^	0,34±0,05^**b**^	--	0,35±0,24
C20:3n6	0,48±0,07^**c, a**^	0,43±0,02^**d, a**^	0,46±0,09^**a**^	0,06±0,04^**b**^
C20:3n3	0,005±0,01^**e, c**^	0,26±0,24^**f**^	0,24±0,31^**d**^	--
C22:1 + 20:4	0,42±0,14^**c**^	0,80±0,43^**d**^	0,71±0,37^**d**^	0,64±0,20^**d**^
C20:5n3	0,005±0,01^**c, e**^	0,035±0,03^**d**^	0,04±0,03^**f**^	0,025±0,03
*SFA*	29,68±0,53^**a**^	32,60±0,75^**b, a**^	29,53±0,74^**b, a**^	40,87±2,98^**b**^
*MUFA*	44,43±1,34^**e, a, c**^	42,34±1,83^**f, c**^	41,05±1,35^**b**, **d, e**^	42,62±1,82^**d, f**^
*PUFA*	25,46±1,21^**c, a**^	24,25±1,33^**d, a**^	28,69±0,87^**b, a**^	15,86±1,97^**b**^
*n-3*	1,32±0,07^**a**^	1,39±0,20^**e, a**^	1,92±0,38^**b, f, a**^	0,54±0,09^**b**^
*n-6*	24,13±1,16^**c, a**^	22,86±1,25^**d, a**^	26,77±0,82^**b, a**^	15,32±1,94^**b**^
*n-6/n-3*	18,24±0,74^**e, a**^	16,75±1,85^**f, c, a**^	14,40±2,88^**f, d, a**^	29,01±5,34^**b**^
*PUFA/SFA*	0,857	0,744	0,97	0,39
*MUFA/SFA*	1,50	1,30	1,39	1,04

n = 12 per Tretman, ^a:b^p<0,001, ^c:d^p<0,05, ^e:f^p<0,01.

**Table 3:** **Fatty Acids Composition (mg/100 g) of Drumstick**$\left(\overset{¯}{X}\pm \;\mathrm{Sd}\right)$

**Fatty acids (range)**	**Tretman**			
	**I**^**a**^	**II**^**a**^	**I**^**b**^	**II**^**b**^
C14:0	0,32±0,01^**a**^	0,33±0,01^**a**^	0,34±0,04^**c**^	0,38±0,03^**b, d**^
C15:0	0,047±0,00^**a**^	0,05±0,02	0,05±0,005^**b, c**^	0,07±0,01^**b, d**^
C16:0	23,03±0,85^**a**^	23,22±1,01^**a**^	21,52±0,63^**b, a**^	23,69±1,36^**b**^
C16:1	4,38±0,36^**c, a**^	4,06±0,48^**d, c**^	3,80±0,22^**b, d, c**^	4,40±0,67^**d**^
C17:0	0,09±0,00^**e**^	0,10±0,01^**f**^	0,09±0,00	0,09±0,02
C18:0	6,00±0,54^**a, c**^	6,93±0,53^**b, a**^	5,96±0,08^**b, e**^	6,90±0,79^**f**^
C18:1c-9	39,89±1,14^**c, e**^	38,69±0,98^**d, c**^	38,13±1,52^**f, c**^	39,26±0,76^**d**^
C18:2n6	22,92±1,39^**a**^	23,03±2,05^**a**^	26,61±1,51^**b, a**^	22,22±1,99^**b**^
C18:3n6	0,18±0,05^**a**^	0,19±0,02^**e, a**^	0,16±0,02^**f, a**^	0,08±0,02^**b**^
C18:3n3	1,27±0,10^**a**^	1,23±0,12^**a**^	1,55±0,12^**b, a**^	1,03±0,35^**b**^
C20:0	0,07±0,03	0,09±0,00^**a**^	0,09±0,01^**a**^	0,06±0,01^**b**^
C20:1	0,47±0,04^**c**^	0,46±0,03^**c**^	0,43±0,06^**d**^	0,36±0,12^**d**^
C20:2	0,24±0,04^**e**^	0,30±0,04^**f, c**^	--	0,39±0,14^**f, d**^
C20:3n6	0,54±0,07^**e, a**^	0,45±0,04^**f, a**^	0,37±0,036^**b**^	0,22±0,04^**b**^
C20:3n3	0,02±0,02	0,07±0,13	0,07±0,15	--
C22:1 + 20:4	0,47±0,08^**a**^	0,60±0,20^**c, e**^	0,39±0,21^**d**^	0,29±0,09^**b, f**^
C20:5n3	--	0,02±0,02^**e**^	0,02±0,02^**e**^	0,12±0,09^**f**^
*SFA*	29,58±1,26^**c, e**^	30,92±1,39^**d, a**^	28,07±0,64^**f, b, a**^	31,60±1,84^**f, b**^
*MUFA*	44,75±1,34^**c, a**^	43,17±1,44^**d**^	42,37±1,61^**b, a**^	44,03±1,08^**b**^
*PUFA*	25,19±1,48^**a**^	25,30±2,15^**a**^	29,17±1,60^**b, a**^	24,07±2,33^**b**^
*n-3*	1,29±0,08^**a**^	1,32±0,14^**a**^	1,72±0,17^**b, a**^	1,15±0,33^**b**^
*n-6*	23,89±1,40^**a**^	23,98±2,06^**a**^	27,45±1,56^**b, a**^	22,92±2,04^**b**^
*n-6/n-3*	18,44±0,53^**a**^	18,31±1,37^**e**^	16,08±1,52^**b, f, e**^	20,84±3,92^**f**^
*PUFA/SFA*	0,85	0,82	1,04	0,76
*MUFA/SFA*	1,51	1,39	1,51	1,39

n = 12 per Tretman, ^a:b^p<0,001, ^c:d^p<0,05, ^e:f^p<0,01.

**Figure 2:**
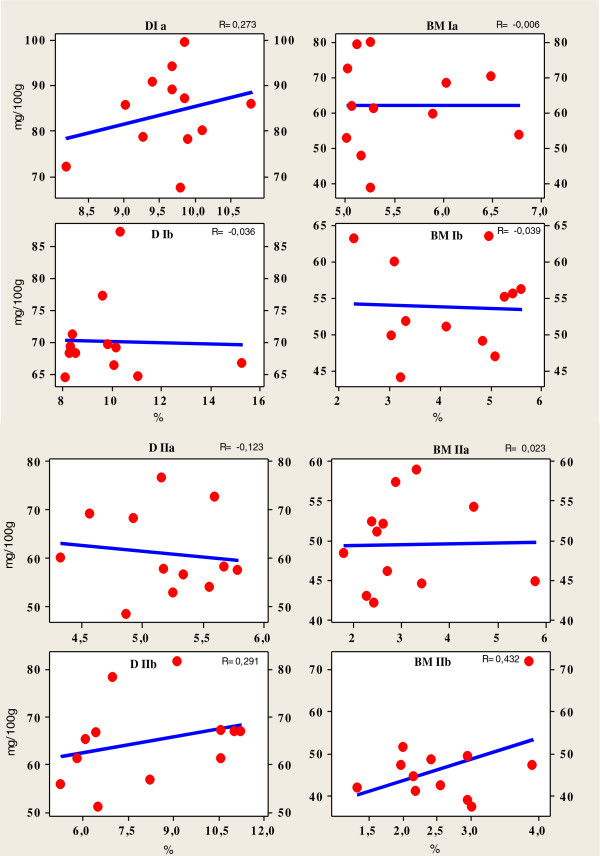
Pearson correlation between content of fats (%) and cholesterol (mg/100 g) in Breast and Drumstick muscle (D -Drumstick, BM - Breast muscle, I, II-farm, a Summer, b Autumn/winter period).

The main fatty acids identified in both the muscles were monounsaturated fatty acid (MUFA) oleic acid (C18:1c9), and highest levels (42.85%) were observed in meat of chicken from farm Ia. In breast muscles, the major saturated fatty acid (SFA) was palmitic acid (16:0) and ranged from 21.35% to 28.53%; and the major polyunsaturated fatty acid (PUFA) was linoleic acid (C18:2n6) and varied from 10.26% to 24.85%. The fatty acid profile of the drumstick muscles, showed a slightly higher fraction of PUFA (C18:2n6) in comparison to SFA (16:0). Although the PUFA/SFA ratio was similar for the four diet groups, n-6/n-3 ratio of meat were significantly different (P < 0.001) between the samples analyzed. This is due to the predominance of corn, soy and sunflower oils the source of fat, which is high in n-6 fatty acids. Comparing breast with drumsticks muscle, the breast contained more SFA, and n-3 PUFA, but less MUFAs, total PUFAs and n-6 PUFA. This effect has been reported by Lopez-Ferrer et al. (1999) [18].

### Roasted meat

The cholesterol concentrations of roasted drumstick and breast muscles are presented in Figure 1B.

The concentration of cholesterol was greater in roasted than in raw meat. The increase in cholesterol correlated well with the observed loss in weight due to water evaporation during roasted (data not shown). The values obtained for the cholesterol varied considerably between samples and between muscles, and thus the values are presented separately (Figure 1B). The drumsticks showed the highest amount, whereas the breast meat presented the lowest values. The cholesterol content showed significant difference (P < 0.001) between the products, varying from 54.13 mg/100 g to 111.64 mg/100 g in the drumsticks versus 53.71 mg/100 g to 99.8 mg/100 g in breast muscles. The total lipid content varied from 8.11% to 15% in drumstick, while in the breast was up to 7%. Pearson correlation coefficients conducted due to water evaporation during roasted, showed moderate significant negative correlation between the content of moisture in raw and content of cholesterol in roasted meat in treatment I, while in treatment II varied from negative (R = -0.236) to strong significant positive correlation (R = 0.638). A similar relationship was observed between the content of cholesterol and fats in roasted meat. The oxidative stability of roasted dark and white chicken meat was studied using the 2-thiobarbituric (TBA) (data not shown). Thigh muscle had higher concentrations (up to 8.63 mg/MAL/kg) of TBA than breast muscle (up to 5.52 mg/MAL/kg). This fact could be explain that thigh muscle had higher concentrations (11.07% - 14.73%) than breast (3.57%-6.25%). There were significant negative interactions between content of TBA, cholesterol and fats in both muscles, except in one case in the breast and drumstick meat, where is a significant positive correlation was noted (R = 0.256 to 0.608).

## Discussion

### Carcass quality

Meat provides a good source of minerals and has a dual role in relation to daily iron supply due to the high bioavailability of haem iron in muscle tissue and the enhancing effect on the bioavailability of non-haem iron [19]. Thus, the percentage of iron that is heme is important in estimating the total bioavailable iron in foods. Heme iron values averaged 29% and 40% for breasts and drumstick chicken meat, respectively [20]. Recently, Wang et al. (2009) [21] found that pre-slaughter exposure to heat reduced the oxidative stability of broiler breast muscle protein, decreasing its functional properties. At the arrival at the abattoir, it has been observed that holding birds at different temperatures can influence some meat quality properties. There are reports of lower pH from birds kept at elevated temperatures compared to broilers held in cooler conditions [22]. McKee and Sams (1997) [23] observed that turkeys subjected to elevated temperatures prior to slaughter exhibited more PSE meat characteristics. Moreover, Warriss et al. (1999) [24] indicated that holding broilers for more than 1 hour prior to slaughter resulted in higher breast pH due to effects of glycogen muscle depletion. Water-holding capacity (WHC) is an important meat quality attribute because color, juiciness, and tenderness are all partially dependent on the ability of the meat to retain moisture under normal storage conditions and during thermal processing [25]. Water-holding capacity is also factor used for evaluating PSE meat. The results from this study indicate that variations in broiler meat pH and Water-holding capacity, could be related to differences in the shelf-life of the product. High muscle pH produced conditions that make dark-colored fillets more susceptible to bacterial spoilage than light-colored fillets when held at the same refrigerated storage conditions. It may be advantageous for industry to separate broiler meat according to color and to divert dark-colored fillets into further processed products where the shelf-life could be extended. However, it should be noted that during the cooking process, fillets that have a high muscle pH may result in a pink, undercooked appearance [26-28].

### Cholesterol concentration

**Figure 3:**
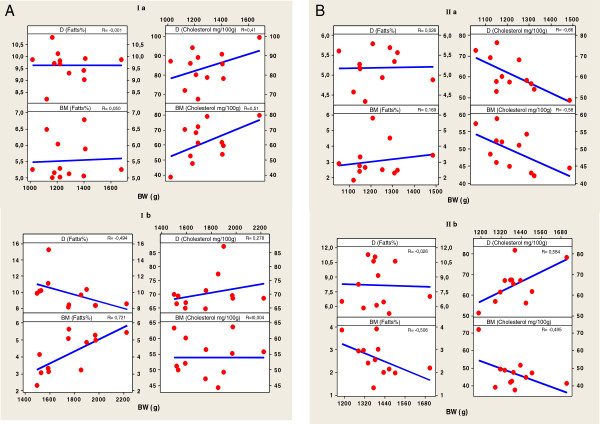
**A. Pearson correlation between Carcass, weight (g), and content of fatts (%) and cholesterol (mg/100 g) (D -Drumstick, B - Breast muscle, I, II-farm, a Summer, b Autumn/winter period). B**. Pearson correlation between Carcass, Breast muscle and Drumstick weight (g), and content of fatts (%) and cholesterol (mg/100 g) (D -Drumstick, B **-** Breast muscle, I, II-farm, a Summer, b Autumn/winter period).

Cholesterol is an important molecule that has roles in membrane structure as well as being a precursor for the synthesis of molecules such as steroid hormones, vitamin D, and bile acids [29]. Furthermore, the lack of correlation or a weak correlation between the fat and cholesterol contents of meat reported in our study (Figure 2), is in line with what has been found in previous studies [30,31]. In regard to the effect of carcasse weight on total lipid and cholesterol content in meat, total lipid content in both muscles was independent of carcasse weight, while cholesterol content varied from moderate significant positive (R = 0.584) to high significant negative correlation (R = -0.660) (Figure 3A, B). The reason of these facts could be explained due to carcasses were taken within a wide range of weight (1021.7 to 2226.7 g) to obtain sufficient variability of total lipid and consequently of cholesterol. It was shown that the subcellular distribution of cholesterol in muscle tissue changed as intramuscular fat increased but, this resulted in no overall change in the total cholesterol in the muscles [30]. A significant factor affecting the cholesterol content of poultry is presence of skin in many retail cuts. Poultry skin has the greatest cholesterol concentration compared with poultry meat or poultry fat. Chicken has also been shown to contain slightly higher cholesterol contents than beef and pork in other studies [30,32]. The differences in cholesterol content among different muscles of the same species and between the same muscles in different species are generally explained by variations in absorption and the biosynthesis of cholesterol, lipoprotein metabolism, diet, muscle fiber type distribution, genetic variation, subcutaneous and intramuscular fat, body weight [13,14,16,33,34], as well as cell size. Cholesterol can be obtained directly from the diet, or it can be synthesized in cells from 2-carbon acetate groups of acetyl-coenzyme A. Because the synthetic pathway is under feedback control of dietary cholesterol, the percentage of cholesterol arising from de novo biosynthesis or from the diet depends upon the amount of cholesterol that is ingested. Even when cholesterol intake is very low, *de novo* biosynthesis will enable the production of the cholesterol required to supply the large variety of biological processes in which this molecule is involved. The human diet nowadays usually includes excessive levels of cholesterol and saturated fat [35]. Various international institutions (e.g., World Health Organization) have drawn up nutritional recommendations that include limitations that refer not only to the amount of fat and the fatty acid composition, but also the cholesterol levels in foods, of which meat and meat products constitute a major part [7].

### Fatty acid composition of breast and drumsticks muscle

Differences in tissue fatty acid profiles could be attributed to different roles of fatty acids in these tissues or to their different contents of phospholipids. PUFA are preferentially incorporated into phospholipids [36] and phospholipids are in higher proportion in muscle fat [37]. This study has clearly demonstrated that decreasing the dietary n6/n3, increases the deposition of desirable n3 and n6 long chain PUFA in the edible tissue, thereby achieving nutritionally enriched meat. Meat has been implicated in imbalanced fatty acid intake by consumers, due to some meats naturally having a low PUFA to SFA ratio (P/S) ratio of around 0.1. Thus a P/S ratio of 0.4 is often recommended [5,38]. Also, it is recommended that dietary energy coming from fat for human consumption should not be more than 30%, while the relation among SFA, MUFA and PUFA should equal 1:1:1 [39]. In the current study the P/S ratio was higher than 0.4, ranging between 0.39 to 0.97 in breast muscle versus 0.76 to 1.04 in drumsticks muscle. Recently, epidemiological studies suggested that the major risk factor for atherosclerosis and coronary heart diseases was found to be a high n-6/n-3 ratio rather than a high intake of cholesterol and the consequent hypercholesterolemia [40]. In the present experiment, the higher n-6/n-3 ratio indicating its potent role in defining the quality of broiler meat. The saturated fatty acids (SFAs) in poultry tissues rely upon their presence in the diet and their synthesis in the liver [41]. The SFAs synthesis is inhibited in the liver more during digestion of unsaturated fats than saturated fats [42]. Also, the increase of PUFAs decreased the synthesis of monounsaturated fatty acids (MUFAs) by inhibiting the activity of 9-desaturase complex, which is the key enzyme needed to convert SFAs to MUFAs [43]. Our results supported recently, researchers that dietary polyunsaturated fatty acids decreased body fat deposition [44]. As noted earlier, the fatty acid composition of meat is influenced by factors other than diet including genotype, gender and age of the animal. Genetic factors affect the meat fatty acid composition, but to a lower extent than dietary factors [45]. Genetic variability relates to differences between species, between breeds or lines, variation due to the crossing of breeds and variation between animals within breeds.

The results of the current study indicated that the fatty acid profiles of broiler tissues may be customized by feeding a diet containing a lipid having the fatty acid composition desired of the resulting tissue. In view of concern over excessive SFA or PFA intakes by humans, possible future applications of these results include increasing the broiler tissue level of a specific fatty acid or mixture of fatty acids thought to be beneficial to human health (e.g., oleic acid or oleic acid plus omega-3 fatty acids). Additionally, manipulation of the tissue content of specific fatty acids may benefit producers of further processed products through better control of fat liquidity and susceptibility to lipid oxidation. This concept will provide broiler producers a means to customize their product more effectively compete with beef in fulfilling the demands of increasingly health-conscious consumers.

### Roasted meat

Chicken meat is particularly susceptible to lipid oxidation as it has a high proportion of unsaturated fatty acids, and the formation of COP during storage or heat-processing.appears to vary depending on the part of the chicken and the cooking method used [8,46]. The levels of COP reported in fresh or freshly processed chicken are generally low or undetectable [8,46]. On the other hand, cooking methods can increase significantly cholesterol oxidation up to 1.5% (COP/cholesterol) in fried chicken patties [14], rising from 4 to 10 times the total COP content in cooked meat than in raw meat [8,46]. Grau et al. (2001) [47] has observed that the dietary lipid sources including beef tallow, fresh and oxidized sunflower oils did not have a considerable effect on the cholesterol content in raw chicken meat, whereas the content of cholesterol oxidation products was significantly higher only in the chickens fed with sunflower oil, reported in our study. Studies in rabbits, chicken, and other susceptible species showed that dietary cholesterol and oxycholesterols can lead to atherosclerosis [48-50]. Cholesterol and cholesterol oxidation products (COPs, oxycholesterols) are also known to be a risk factor for coronary heart disease (CHD) and COPs may have cytotoxic, mutagenic and atherogenic effects, and thus are harmful to humans [51].

## Conclusion

In conclusion, chicken meat is characterized by high nutritive value. Due to low cholesterol, a fairly high protein and PUFA content, it can be used as a valuable inclusive component of the human diet, with potential health benefits. It is evident from this review of the scientific literature that additional research is required in several areas, notably: (1) the true impact of dietary fatty acid supplementation on animal health and welfare; (2) the impact of fatty acid enrichment of animal diets on production costs; (3) the effect of fatty acid changes on product shelf-life and quality.

In view of concern over excessive SFA or PFA intakes by humans, possible future applications of these results include increasing the broiler tissue level of a specific fatty acid or mixture of fatty acids thought to be beneficial to human health (e.g., oleic acid or oleic acid plus omega-3 fatty acids). Additionally, manipulation of the tissue content of specific fatty acids may benefit producers of further processed products through better control of fat liquidity and susceptibility to lipid oxidation.

## Methods

### Experimental site

The experiment was conducted on the (farm I- 45°44′N, 20°72′ E, 76 m, farm II- 45°52′N, 19°27′ E, 99 m) province of Vojvodina, northern part of Serbia.

### Birds, management, and diets

During the experimental period from June to November of 2012, and February of 2013, two homogeneous groups of male and female (50:50%) mainly Ross 308 and partially Hubbard broilers, were reared under commercial conditions in two separate houses (20 000 animals per house) and were fed *ad libitum* a commercial diet. When the broilers were 38 days old, they were slaughtered at a commercial abattoir. Prior to slaughter, broiler was subjected to a total feed withdrawal of 8-12 h, including a holding time at the processing plant of 2-3 h.

### Slaughter procedures

On day 38, the broilers, were stunned individually in a waterbath stunner (2,7 A, 45 V, 380 Hz, 25 s), slaughtered and bled at a local slaughterhouse, using standard industry procedures. After slaughtering and dressing, hot carcasses were chilled two hours at 4°C. Carcasses (n = 12 per farm), were randomly collected and used for subsequent meat quality evaluation during the summer (n = 24) and winter (n = 24) season.

### Sample collection

After chilling, all carcasses were placed in insulated polystyrene boxes on ice and transferred to the laboratory within 2 h of chilling. Then, carcasses were weighted and refrigerated for 24 h. The breast with skin (*m. pectoralis major*) and drumstick meat with skin (muscles of regio tibio-femoralis) were cut and separated. Whole samples of both muscles (about 20 g) were placed in open aluminium pans and roasted in an electric oven (pre-heated to 220°C) for 30 min. Total lipid and cholesterol content were determined in raw and roasted breast and drumstick meat with skin.

### Analytical procedures

#### *Fatty acids*

Total lipids for fatty acids determination in meat samples, were extracted from the sample by accelerated solvent extraction (ASE 200, Dionex, Sunnyvale, CA) with a mixture of n- hexane and isopropanol (60:40, v/v) in 33 ml extraction cell at 100°C and nitrogen pressure of 10.3 MPa [52]. The solvent was removed under the stream of nitrogen at 50°C until dryness in solvent evaporator (SE 500, Dionex, Sunnyvale, CA). The fat extract was further used for fatty acids determination.

Total lipids were further converted to fatty acid methyl esters (FAMEs) by using 0.25 M trimethylsulfonium hydroxide (TMSH) in methanol [53]. FAMEs were determined by capillary gas chromatography on GC Shimadzu 2010 (Kyoto, Japan) equipped with flame ionization detector and capillary HP-88 column (100 m × 0.25 mm × 0.20 μm, J&W Scientific, USA). Separation and detection were performed under the following temperature program: initial temperature 125°C, rate 10°C min-1 to 175°C, hold 10 min, rate 5°C min-1 to 210°C, hold 5 min, rate 2°C min-1 to final temperature of 230°C, hold 12 min. Total analysis time was 50.5 min. The injector and detector temperatures were 250°C and 280°C, respectively; split ratio 1:50; volume 1 μL; carrier gas, N2, 1.33 mL/min; make-up gas, N2, 30 mL/min; detector gases, H2, 40 mL/min; synthetic air, 400 mL/min. The chromatographic peaks in the samples were identified by comparing relative retention times of FAME peaks with peaks in Supelco 37 Component FAME mix standard (Supelco, Bellefonte, USA).

Moisture and ash content of meat samples were determined according to the ISO methods [54,55]. Nitrogen was measured according to the Kjeldahl method (Tecator-Kjeltec system 8400, Tecator Foss, Sweden) and protein was calculated as N × 6.25. Total lipid content was determined by extraction of fat by petroleter (Soxhlet) after acid hydrolysis of samples [56]. All measurements were performed in triplicate.

#### *pH*

Muscle pH was determined using an Testo® 230 meter (Lenzkirch, Germany), fitted with a combined glass electrode, previously calibrated at pH 4.0 and 7.0 with standard buffers (Mallinckrodt Chemicals, Phillipsburgh, NJ, USA) stored at room temperature (20°C). The pH values were determined by inserting the probe into the geometric center of the thickest part of the muscles. The average pH value was defined through 3 measurements of the same area, and the procedures used for determination were the same for all of the samples. The samples were stored at 4°C before measuring the pH value at 24 h postmortem.

#### *Water-holding capacity*

Water-holding capacity of muscle samples were performed using the methodology of Wierbicki and Deatherage (1958) [57]. Briefly, 300 ± 5 mg of fresh, muscle tissue were weighed onto a piece of Whatman No. 1 filter paper, which had been stored in a desiccator over saturated potassium chloride. The sample was then pressed at 345 N/cm^2^ for 1 min. Areas of the meat and moisture were traced and subsequently measured using a compensating planimeter (Planix 8; Sokkia Corp., Overland Park, KS, USA). All samples were run in duplicate, and the percentage of free water was calculated by the following equation: free water (%) = (((moisture surface area - meat surface area) × 61.1)/total moisture) × 100, whereas the percentage of bound water was calculated by subtracting the free water from 100.

#### *Cholesterol determination*

Cholesterol determination in the meat was determined after direct saponification (without prior lipid extraction) according to the method described by Maraschiello et al*.* (1996) [58] and followed by HPLC analysis; the data are expressed as mg/100 g fresh meat. To ca. 100 mg of each homogenized chicken muscle sample 2 ml of 0,5 M KOH in methanol was addeded and tubes were vortexed for 30 s. The mixture was directly saponified at 80°C during 1 h. After cooling, 2 mL of distilled water, saturated with NaCl was added. The tubes were vortexed for 30s followed by addition of 3 mL diethilether/hexane (1:1, v/v) and centrifugated for 10 min at 300 g. The upper phase was transferred to a clean tube and the ether/hexane extraction step was repeated twice. All three extracts were combined and evaporated to dryness under stream of nitrogen. The dry extracts were dissolved in 1000 μl of mobile phase used for HPLC analysis and then immediately filtered: 10 μl was injected into HPLC. Cholesterol determination in the extract (from direct saponification, describe above) was performed by using HPLC/PDA system (Waters 2695 Separation module/Waters photodiode array detector, USA), on a Phenomenex Luna C 18 reverse/phase column, 150 mm × 3,0 mm, 5μm particle size with C18 analytical guard column, 4.0 mm × 2.0 mm, at room temperature. The injected volume was 10 μL The mobile phase was isopropanol-acetonitrile (20:80, v/v) at a flow rate of 1.2 ml min ^-1^, isocraticaly. Detection was performed at 210 nm. Total analysis time lasted 10 min. Quantification of cholesterol was done by external standardization in a linear concentration range from 25 mg/100 g to 125 mg/100 g. Recoveries of the spiked quantities ranged from 66.30 to 74.80%. Empower Pro software was used to control the HPLC system as well as for data acquisition and data processing.

#### *Iron determination*

Determinations of Fe in meat samples were performed by flame atomic absorption spectrophotometry (Analyst 300, Perkin Elmer, USA) following the methods described by Jorhem (2000) [59]. All reagents were of analytical grade, and MilliporeMilliQ deionized water was used throughout the procedures. Glass and polyethylene materials were soaked in HNO_3_ (Merck, Argentina) and washed with deionized water. Standard solutions of Fe (Perkin Elmer, USA) were prepared immediately before use by dilution (with deionized water) of a 1000 mg/l standard solution. Quality control was performed by running bovine liver standard (NIST standard, SRM 1577c, Gaithersburg, USA). Iron contents was expressed in mg/kg of wet tissue.

#### *Statistical analysis*

The statistical analysis was performed with a Minitab 16.1.0.0 (Minitab Inc. © USA). The farms was used as the experimental unit for all the analyses done. Data are presented as mean ± standard deviation (SD) for each treatment. For comparison between treatments, data were analyzed for normal distribution by Kolmogorov–Smirnov and equal variance by Kruskal–Wallis One Way Analysis of Variance (ANOVA procedure, SAS Inst. Inc., Cary, NC). When significant differences were identified among treatment means, they were separated using Tukey’s Honestly Significant Difference test (P <0.05).

## Abbreviations

HPLC/PDA: HPLC Photo Diode Array; CVD: cardiovascular disease; SFA′s: saturated fatty acids; UFA: unsaturated fatty acids; PUFA′s: polyunsaturated fatty acids; TBARS: Thiobarbituric acid reactive substances; WHC: Water-holding capacity; PSE: Pale, soft, and exudative.

## Competing interests

The author’s declare that they have no competing interests.

## Authors’ contributions

DM completed the project, established the study, analyzed the data, wrote the manuscript, and contributed to reviewing/editing the manuscript. DV, ZM and JNT have made substantial contributions to conception and design of the study, participated in the discussion and critically revised the manuscript. DT, NP and ZP performed statistical analyse, contributed to assay, researched and evaluated the literature. All authors read and approved the final manuscript.
